# Transport processes of the legume symbiosome membrane

**DOI:** 10.3389/fpls.2014.00699

**Published:** 2014-12-15

**Authors:** Victoria C. Clarke, Patrick C. Loughlin, David A. Day, Penelope M. C. Smith

**Affiliations:** ^1^School of Biological Sciences, University of Sydney, Sydney, NSW, Australia; ^2^School of Biological Sciences, Flinders University, Adelaide, SA, Australia

**Keywords:** legume, rhizobia, symbiosis, transport, membrane

## Abstract

The symbiosome membrane (SM) is a physical barrier between the host plant and nitrogen-fixing bacteria in the legume:rhizobia symbiosis, and represents a regulated interface for the movement of solutes between the symbionts that is under plant control. The primary nutrient exchange across the SM is the transport of a carbon energy source from plant to bacteroid in exchange for fixed nitrogen. At a biochemical level two channels have been implicated in movement of fixed nitrogen across the SM and a uniporter that transports monovalent dicarboxylate ions has been characterized that would transport fixed carbon. The aquaporin NOD26 may provide a channel for ammonia, but the genes encoding the other transporters have not been identified. Transport of several other solutes, including calcium and potassium, have been demonstrated in isolated symbiosomes, and genes encoding transport systems for the movement of iron, nitrate, sulfate, and zinc in nodules have been identified. However, definitively matching transport activities with these genes has proved difficult and many further transport processes are expected on the SM to facilitate the movement of nutrients between the symbionts. Recently, work detailing the SM proteome in soybean has been completed, contributing significantly to the database of known SM proteins. This represents a valuable resource for the identification of transporter protein candidates, some of which may correspond to transport processes previously described, or to novel transport systems in the symbiosis. Putative transporters identified from the proteome include homologs of transporters of sulfate, calcium, peptides, and various metal ions. Here we review current knowledge of transport processes of the SM and discuss the requirements for additional transport routes of other nutrients exchanged in the symbiosis, with a focus on transport systems identified through the soybean SM proteome.

## INTRODUCTION

Nitrogen is an essential macronutrient for plants and can be a limiting factor in crop growth. Nitrogen fertilizer is often used to supplement soils and produce high quality yields, but production of commercial quantities of nitrogen fertilizer is energy-expensive and the fertilizer can have negative effects on the environment, causing pollution of ground water (Vance, 2001). A natural alternative to commercial fertilizers is symbiotic nitrogen fixation in legumes, which can reduce the need for nitrogen fertilizer and can boost nitrogen reserves in the soil.

Biological nitrogen fixation (BNF) by rhizobia in legume nodules is responsible for the addition of approximately 40 million tons of nitrogen to agricultural systems each year (Herridge et al., 2008). As well as providing a protein-rich food source for humans and animals, legume crops are used to enrich soil nitrogen reserves, enabling growth of other crop species (Oldroyd et al., 2011). BNF also has the added advantage of decreased environmental impacts compared to synthetic nitrogen fertilizers, and subsequent reduction in costs associated with crop production (Udvardi and Poole, 2013).

Due to its importance, the legume:rhizobia symbiosis has been the focus of much research, with the ultimate aim to improve existing symbioses and potentially expand BNF into other non-legume crop species, such as cereals (Beatty and Good, 2011).

## BIOLOGICAL NITROGEN FIXATION

Biological nitrogen fixation occurs through activity of the enzyme nitrogenase, which is found only in certain prokaryotes, including those of the Rhizobiaceae family (or rhizobia). The enzyme converts atmospheric nitrogen to ammonium, a plant-available form of nitrogen, but requires large amounts of ATP to fuel the conversion (Halbleib and Ludden, 2000). Legumes, such as soybeans, are able to form an association with nitrogen-fixing soil bacteria of the Rhizobiaceae family (termed rhizobia). In this symbiotic relationship, atmospheric nitrogen is fixed by the bacteria and made available to the plant in exchange for organic acids and other nutrients (Lodwig and Poole, 2003). This mutually beneficial association occurs within specialized root structures termed nodules.

## ROOT NODULE FORMATION

The relationship between rhizobia and their legume hosts begins with the exchange of signals between the symbionts. Free-living rhizobia are attracted to legume roots through the exudation of phenolic flavonoid compounds (Hirsch, 1992). Flavonoid perception by rhizobia triggers the synthesis of Nod factors, symbiosis-specific lipochitooligosaccharide compounds which activate nodule organogenesis and induce cellular changes in the plant roots to facilitate bacterial infection (Oldroyd and Downie, 2004). The recognition of Nod factors leads to a series of morphological changes in receptive plant root cells including root hair deformation and the initiation of the infection thread through which the rhizobia travel into the root cortical cells. In the *Medicago truncatula*:*Sinorhizobium meliloti* symbiosis, two flotillins (lipid raft markers) are essential for infection thread initiation, suggesting this initiation process involves lipid rafts on the root cell plasma membrane. Infection results in polarized root-hair tip growth, invagination of the plant cell membrane and the formation of the nodule meristem (Timmers et al., 1999; Esseling et al., 2003). The formation of the nodule meristem in legumes can give rise to two distinct patterns of nodule development, determinate and indeterminate growth. Indeterminate nodules are characterized by a tip-growing meristem as opposed to the transient meristem present in determinate nodules (Oldroyd et al., 2011).

Once inside the cortical cells, the rhizobia divide and multiply, and these cells are now termed infected cells. As the infected cells expand inside the growing nodule, the rhizobia are released from the infection thread into vesicles termed symbiosomes (Roth et al., 1988). This was initially thought to be an endocytotic process, and indeed, the endosomal marker Rab7 is present on mature symbiosomes (Limpens et al., 2009). More recent studies, however, have demonstrated that exocytotic vesicle-associated membrane proteins are required during the formation of the symbiosis, suggesting rhizobial release into symbiosomes is an exocytotic process (Ivanov et al., 2012).

The symbiosome is surrounded by a membrane of plant origin known as the symbiosome membrane (SM) which is derived from the infected cell plasma membrane, but becomes specialized in its role to contain the rhizobia (Whitehead and Day, 1997). Within the symbiosome in determinate nodules, rhizobia continue to multiply before differentiating into bacteroids, the symbiotic form of rhizobia in which symbiosis-related genes are induced (Whitehead and Day, 1997). Mature symbiosomes result from the coordinated division of bacteria and growth of the surrounding SM.

## THE SYMBIOSOME MEMBRANE

The SM surrounds one or more differentiated bacteroids, effectively excluding them from the plant cytosol. The region between the SM and the bacteroids is termed the symbiosome space (SS). The SM is a selectively permeable physical barrier between plant and bacteroid, representing a regulation point under plant control for the movement of solutes between symbionts. The SM is therefore proposed to contain an array of transporters and channels to facilitate this (Whitehead and Day, 1997).

After its initial formation, the SM undergoes enormous proliferation to enable it to accommodate the dividing bacteroids (Roth and Stacey, 1989). It is estimated that the SM surface area in an infected cell is up to one hundred times that of the plasma membrane (Roth and Stacey, 1989). Protein trafficking and secretion have important roles in the symbiosis, as the expanding SM requires the synthesis of large amounts of lipids and proteins to meet the increasing requirements for SM in the infected cell. The SM composition varies throughout the existence of the symbiosome to facilitate the different transport requirements of the symbionts (Whitehead and Day, 1997).

Several proteins have been identified which possess an N-terminal signal sequence directing them to the symbiosome (Liu et al., 2006; Hohnjec et al., 2009; Meckfessel et al., 2012). For example, the SS localized N-terminal region of *M. truncatula* nodulin 25 (MtNOD25) contains a signal peptide that can drive symbiosome targeting of heterologously expressed proteins, and this signal sequence is conserved across several other symbiosome proteins (Hohnjec et al., 2009). MtENOD8, a SS localized protein (Coque et al., 2008), also contains a signal peptide which directs subcellular targeting (Meckfessel et al., 2012) but it also contains an internal sequence directing it into the symbiosome, without the requirement for the N-terminal signal peptide (Meckfessel et al., 2012). However, an N-terminal signal sequence is not present in all proteins targeted to the symbiosome, suggesting additional trafficking pathways exist (Catalano et al., 2004).

## IDENTIFYING SYMBIOSOME MEMBRANE TRANSPORTERS

Analysis of rhizobia mutants for transport processes and their N-fixation phenotype has been a useful method to identify the compounds transported across the SM (Udvardi and Day, 1997; Udvardi and Poole, 2013). SM transport proteins in soybean, *M. truncatula*, *Lotus japonicus* and other legumes have been identified using a range of biochemical and molecular approaches. *M. truncatula* forms indeterminate nodules, while soybean and *L. japonicus* both form determinate nodules. As well as differences in nodule meristem development, each species has distinct advantages for particular applications. Both *L. japonicus* and *M. truncatula* have small diploid genomes and a short life cycle well suited to genetic analysis. While soybean has a larger, polyploid genome, its larger and more numerous nodules allow easier extraction and isolation of symbiosomes and their components. Soybean is an agriculturally important legume and its genome has been fully sequenced (Schmutz et al., 2010).

The publication of the soybean genome, together with advance in sequencing technologies have enabled high-resolution transcriptome studies to be undertaken. Two soybean transcriptome atlases have been produced, detailing gene expression profiles in different soybean tissues including roots and nodules (Libault et al., 2010; Severin et al., 2010) and gene expression atlases are available for *L. japonicus* (Verdier et al., 2013) and *M. truncatula* (Benedito et al., 2008). These transcriptome databases provide a comprehensive resource for the identification of tissue-specific gene expression.

Recently, the combination of resources in soybean, together with the relative ease of SM protein extraction, has allowed the generation of a proteome of the soybean SM from *Bradyrhizobium japonicum* infected plants (Clarke et al., unpublished). This provides a rich resource of novel candidate SM transport proteins and here we discuss our current understanding of the transport processes of the SM as well as the potential roles for these recently identified proteins in the symbiosis.

## TRANSPORT ACROSS THE SYMBIOSOME MEMBRANE

**FIGURE 1:**
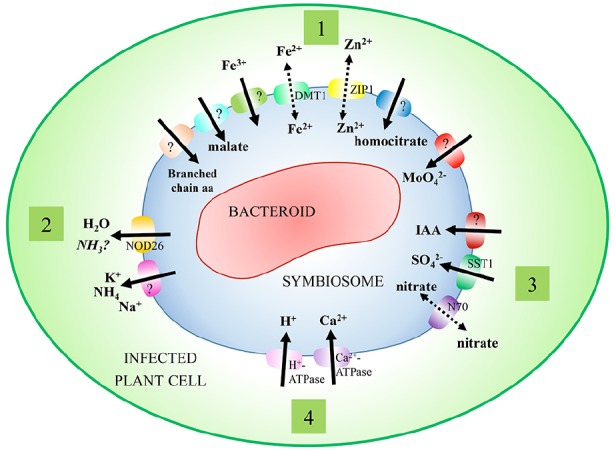
**Characterized transport processes of the symbiosome membrane.** Symbiosomes exist within infected plant cells, where they act to partition nitrogen-fixing bacteroids from the cell cytosol. A range of transport processes have been characterized on the symbiosome membrane to facilitate movement of solutes between symbionts. These include (1) transport processes supporting the primary needs of symbionts (nitrogen, malate, and metal ions), (2) efflux processes (nitrogen), (3) secondary transport processes (nitrate, sulfate, and IAA), and (4) regulatory transport processes (H^+^-ATPase, calcium, and water flux).

The SM is an interface that regulates the distribution of metabolites between the host plant and nitrogen-fixing bacteroids. The presence of transporters and channels on this membrane, whose activity and specificity can be tightly regulated, allows for the controlled distribution of metabolites and signaling molecules. Early work using biochemical assays with isolated symbiosomes provided the groundwork on which molecular and genomic research has expanded, although genes encoding many of these transport processes have not yet been identified (Figure 1; Table 1).

**Table 1:** A summary of characterized transport processes of the legume symbiosome membrane indicating transported substrate, corresponding gene (if identified), and related publication(s).

**Compound transported**	**Biochemical characterization**	**Gene encoding transporter (evidence)**	**Reference**	**Direction**
Malate	Monovalent anion uptake assays	Not identified	Udvardi et al. (1988)	Import
Fe^2+^	Ferrous iron uptake into symbiosomes (radioactive assay); ferrous iron uptake into yeast (equivalent to efflux from symbiosome)	*DMT1*? (yeast complementation)	Moreau et al. (1995, 1998), Kaiser et al. (2003)	Bidirectional?
Fe^3+^	Ferric chelate uptake assay	Not identified	LeVier et al. (1996)	Import
Zn^2+^	Zn uptake into symbiosomes and yeast (radioactive assay)	*ZIP1* (yeast complementation)	Moreau et al. (2002)	Bidirectional?
Homocitrate	Inferred	Not identified	Hoover et al. (1989), Hakoyama et al. (2009)	Import
IAA	IAA uptake assay into symbiosomes	Not identified	Rosendahl and Jochimsen (1995)	Import
SO_4_^2–^	No biochemical activity measured on symbiosomes	*SST1* (yeast complementation)	Krusell et al. (2005)	Import?
Nitrate	Anion uptake assay	*N70* (expression in *Xenopus* oocytes, voltage clamp)	Udvardi et al. (1991), Vincill et al. (2005)	Bidirectional?
Molybdate	Inferred	Not identified	Delgado et al. (2006)	Import
Ca^2+^	ATP-dependent Ca^2+^ uptake	*Ca^2+^-ATPase* (sequence homology, localization)	Andreev et al. (1999), Krylova et al. (2012), Clarke et al. (unpublished)	Import
H^+^	Assays for P-type H^+^-ATPase	*H^+^-ATPase* (sequence homology, localization)	Blumwald et al. (1985), Udvardi et al. (1991), Fedorova et al. (1999), Wienkoop and Saalbach (2003), Clarke et al. (unpublished)	Import
Branched chain amino acids	Inferred	Not identified	Prell et al. (2009)	Import
Amino acids?		*GmAPC1* (sequence homology, localization)	Clarke et al. (unpublished)	?
K^+^, Na^+^, NH_4_^+^	Patch-clamp of voltage-gated monovalent cation channel	Not identified	Tyerman et al. (1995)	Export
H_2_O, NH_3_?	H_2_O, NH_3_ movement across the SM	*NOD26* (reconstitution into proteoliposomes, expression in *Xenopus* oocytes)	Fortin et al. (1987), Rivers et al. (1997), Dean et al. (1999), Niemietz and Tyerman (2000), Hwang et al. (2010)	Export
Substrate not known		*GmNPF family members*	Clarke et al. (unpublished)	?
Substrate not known		*GmPCR1.1, 1.2*	Clarke et al. (unpublished)	?

It should be noted that the orientation of the symbiosome is such that uptake into the symbiosome is equivalent to efflux across the plasma membrane. Uptake into heterologous expression systems such as yeast or oocyte represents the reverse direction; that is, efflux from a symbiosome. Hence demonstration of uptake into symbiosomes and yeast suggests that a transporter can operate bidirectionally.

### TRANSPORT OF REDUCED CARBON

The primary nutrient exchange across the SM is the transport of a carbon energy source from plant to bacteroid in exchange for fixed nitrogen. BNF is an energy expensive process which requires large amounts of ATP to fuel the reduction of N_2_ (Halbleib and Ludden, 2000). The host plant provides this from photosynthetic products that are oxidized in the bacteroids to generate ATP. This carbon source is derived from sucrose that is transported from source leaves through the phloem to the nodules (Udvardi and Day, 1997). Dicarboxylates are then transported across the SM to the bacteroids (Day et al., 1995). Assays with isolated soybean symbiosomes identified a carrier for monovalent dicarboxylate ions that had a higher affinity for malate than for succinate, suggesting that malate is the major form transported. The existence of this carrier was first demonstrated in soybean in 1988 (Udvardi et al., 1988; Udvardi and Day, 1997), but the protein that transports dicarboxylates has yet to be identified on the SM of any legume.

In the symbiosis-forming non-legume *Alnus glutinosa*, the AtNPF6.3 homolog, AgDCAT1, was identified and shown to transport dicarboxylates when expressed in *E. coli*, although its closest homologs have been characterized as nitrate transporters (Jeong et al., 2004). DCAT1 is a member of the nitrate transporter/peptide transporter (NRT/PTR) family (NPF; Léran et al., 2014). Members of this family are candidates for the dicarboxylate transporter in legumes as transcriptome data from a number of legume species has indicated that NPF encoding genes are induced strongly in nodules (Colebatch et al., 2004; Benedito et al., 2010; Libault et al., 2010; Severin et al., 2010). A number of members of the family were also identified in the soybean SM proteome (Clarke et al., unpublished). However, as this family also includes proteins that transport a range of different compounds including nitrate, auxin, glucosinolate, and peptides (see below), experimental characterization of the substrates will need to be performed before their role can be confirmed.

### TRANSPORT OF AMMONIA AND AMMONIUM

The product of nitrogenase in bacteroids is ammonia, which is thought to diffuse out of the bacteroids into the acidic SS, where much of it is protonated to ammonium (Day et al., 2001a). Reuptake of ammonium into the bacteroids is prevented through repression of the bacteroid ammonium carrier during differentiation of rhizobia to their symbiotic state (Howitt et al., 1986). The SM is energized by an H^+^-ATPase that pumps H^+^ into the SS. Thus a concentration gradient is established that promotes the efflux of NH_3_/NH_4_^+^ into the plant cytosol, where it is rapidly assimilated. In isolated symbiosomes, two avenues have been identified for the transport of NH_3_/NH_4_^+^ across the SM, a channel for facilitated diffusion of ammonia (Niemietz and Tyerman, 2000), and a voltage-gated monovalent cation channel that transports NH_4_^+^ as well as potassium and sodium ions (Tyerman et al., 1995). The movement of NH_4_^+^ is coordinated by the generation of a membrane potential across the SM by the H^+^-ATPase (Udvardi and Day, 1997, see below). The SM protein encoding the monovalent cation channel has not been identified, but nodulin 26 (NOD26), an aquaporin that transports water (Fortin et al., 1987; Dean et al., 1999; Niemietz and Tyerman, 2000; Hwang et al., 2010), is the likely candidate for passage of NH_3_. NOD26 was first identified as an integral membrane protein of the soybean SM (Fortin et al., 1987) and is a member of the major intrinsic protein/aquaporin (MIP/AQP) channel family (Wudick et al., 2009). It is estimated to constitute 10% of the protein content of the SM (Weaver et al., 1994; Rivers et al., 1997), is exclusively localized to the SM, and due to its prevalence is widely used as a marker for the membrane. NOD26 functions as a multifunctional aquaglyceroporin, with *Xenopus* oocyte studies showing it can facilitate the movement of glycerol and formamide (Rivers et al., 1997; Dean et al., 1999). Other studies have shown that it can also facilitate ammonia transport across the SM (Hwang et al., 2010) and it acts as a docking station for cytosolic glutamine synthetase (Masalkar et al., 2010). This localization of glutamine synthetase would promote rapid assimilation of ammonia, thereby creating a strong sink for further export. The detection of both glutamine synthetase and NOD26 in the soybean SM proteome (Clarke et al., unpublished) provides further support for their suggested roles in ammonia release from the symbiosome (Hwang et al., 2010; Masalkar et al., 2010).

### ADDITIONAL TRANSPORT PROCESSES

#### Energization of the SM

The active pumping of H^+^ ions by an H^+^-ATPase generates a proton gradient across the SM, which is thought to drive many other transport processes (Blumwald et al., 1985; Udvardi and Day, 1989; Andreev et al., 1999; Fedorova et al., 1999), as well as the conversion of ammonia to ammonium. P-type H^+^-ATPases are considered to have an important role in the development of the symbiotic association through their acidification of the SS, but they also energize the SM by establishing an electrochemical gradient across the membrane that is necessary for the secondary transport of other solutes (reviewed in Day et al., 2001a). Three related P-type H^+^-ATPases were identified in the soybean SM proteome (Clarke et al., unpublished), while a P-type H^+^-ATPase has been immunolocalized on the SM of soybean (Fedorova et al., 1999) and detected on the SM by proteomic analysis in *L. japonicus* and *M. truncatula* (Wienkoop and Saalbach, 2003; Catalano et al., 2004). Interestingly, the related V-type ATPases have also been identified proteomically on the SM in pea and *L. japonicus* (Saalbach et al., 2002; Wienkoop and Saalbach, 2003), but could not be detected by immunolocalization on the soybean SM (Fedorova et al., 1999). The absence of V-type ATPases in the soybean SM proteome, together with the results of Fedorova et al. (1999), suggest that soybeans may differ from other legumes in their SM ATPase requirements.

#### Calcium transport

It has been suggested that symbiosomes may behave as calcium stores in infected cells (Andreev et al., 1999). Calcium uptake is an active (ATP-driven) process and an ATP-driven Ca^2+^-pump has been characterized on the SM (Andreev et al., 1999; Krylova et al., 2012). Three Ca^2+^-ATPases were identified in the soybean SM proteome (Clarke et al., unpublished). Both the P-type H^+^-ATPases and these Ca^2+^-ATPases are expressed broadly across soybean tissues (Libault et al., 2010; Severin et al., 2010), suggesting recruitment to a new role and location as part of the symbiosis. Whether the symbiosome functions as a calcium store *in vivo* remains to be seen. The presence of a calcium-dependent protein kinase on the SM and its role in malate uptake into the symbiosome (Ouyang et al., 1991; Weaver et al., 1991) certainly shows the potential for calcium to be a key regulator of symbiosome function.

#### Transport of other nitrogenous compounds

Transport of nitrogenous compounds is of general interest in legumes, especially as nodule development is suppressed in the presence of nitrate (Streeter and Wong, 1988). In all plants, nitrogen plays an important regulatory role, which includes lateral root formation and, in the case of legumes, nodulation.

A nitrate transporter, GmN70, has been identified on the SM in soybean (Vincill et al., 2005). The *L. japonicus* ortholog, LjN70, also transports nitrate and both proteins are members of the major facilitator superfamily (MFS; Vincill et al., 2005). It is postulated that these transporters may aid in the regulation of ion and membrane potential through their transport of nitrate, which is known to regulate the symbiosis (Udvardi and Day, 1989). In isolated symbiosomes, rapid uptake of nitrate disperses the membrane potential (Udvardi et al., 1991).

As mentioned earlier, the NPF family has members whose expression is up-regulated during nodule development in a number of legumes. This large family has recently been divided into eight sub-families, NPF1–8 (Léran et al., 2014). Members of the NPF transport a range of nitrogen-based compounds (Williams and Millar, 2001). AtNPF6.3 (AtNRT1.1, CHL1), one of 53 proteins in the NPF of *Arabidopsis*, can transport nitrate (Tsay et al., 2007) and auxin (Krouk et al., 2010), as can the *M. truncatula* homolog MtNRT1.3 (Beeckman and Friml, 2010; Morere-Le Paven et al., 2011). Uptake of the auxin, IAA, by isolated pea symbiosomes has been reported (Rosendahl and Jochimsen, 1995). NPF proteins with dual transport functions are implicated in nutrient sensing roles within the plant, in addition to high- and low-affinity nitrate uptake (Krouk et al., 2010). Other members of the NPF in *Arabidopsis* transport the defense compounds, glucosinolates, in seeds (Nour-Eldin et al., 2012), abscisic acid, peptides and dicarboxylates (see above).

*Medicago truncatula* MtNPF1.7 (previously called LATD/NIP), a nitrate transporter, is essential for the development and maintenance of lateral roots and release of rhizobia into the symbiosome (Bright et al., 2005; Harris and Dickstein, 2010; Yendrek et al., 2010). Complementation using *Chl1* (*NPF6.3*) from *Arabidopsis*, a dual nitrate/auxin carrier, was able to rescue the *latd/nip1* phenotype in lateral root development, but not the phenotype observed in nodules (Bagchi et al., 2012), suggesting it has other, unidentified functions. A member of the same sub-family, GmNPF1.2, is expressed specifically in nodules (Severin et al., 2010) and is present on the SM (Clarke et al., unpublished).

Other proteins homologous to the *Arabidopsis* NRT/PTR Family (NPF) have also been identified in the soybean SM proteome. One of these is closely related to *Arabidopsis* plasma membrane peptide transporters PTR1 and PTR5 (NPF8 sub-family), while others are in the sub-family of PTR3 (NPF5), also identified as a peptide transporter. The relevance of peptide transport on the SM is not clear. However, all these proteins contain the FING motif that is thought to be essential for peptide transport (Stacey et al., 2002). The genes encoding these proteins are expressed specifically in nodules in soybean (Severin et al., 2010) and proteins homologous to GmNPF5.24 and GmNPF5.25 were identified in the *L. japonicus* SM proteome (Wienkoop and Saalbach, 2003).

If NPF members transport peptides into the symbiosome they could be used to lift symbiotic auxotrophy for branched chain amino acids that was recently identified by Prell et al. (2009) in the pea: *Rhizobium leguminasarum* symbiosis (see below).

#### Amino acid transport

Isolated soybean symbiosomes are only weakly permeable to amino acids (Whitehead et al., 1998) so it is not likely that they are major contributors of carbon to bacteroids in soybean (Day et al., 2001b). However, recent studies have demonstrated that pea bacteroids are auxotrophs for branched chain amino acids (Prell et al., 2009). As the enclosed bacteroids are effectively organelles relying on the plant host to synthesize and provide branched chain amino acids, an SM amino acid transporter could act to facilitate this transport. A putative amino acid transporter (GmAPC1) with homology to the acid-polyamine organocation (APC) superfamily was identified in the soybean SM proteome (Clarke et al., unpublished). This family includes members that function as solute:cation symporters and solute:solute antiporters (Wong et al., 2012).

#### Sulfur and molybdenum transport

Sulfur is a component of the metalloclusters of nitrogenase, essential for the reduction of nitrogen, and must be actively transported across membranes (Krusell et al., 2005). LjSST1 was identified from a *fix^–^* mutant in *L. japonicus* and complemented a yeast strain deficient in sulfate transport (Krusell et al., 2005). Peptides matching this sulfate transporter have been identified on the *L. japonicus* SM (Wienkoop and Saalbach, 2003) and two homologs found on the soybean SM (Clarke et al., unpublished). Krusell et al. (2005) reported that *LjSST1* expression is essential for symbiotic nitrogen fixation; knockout mutants grow normally in non-symbiotic conditions but are unable to produce functioning nodules when inoculated with *M. loti.* Studies using ^35^SO_4_^–^ and isolated soybean symbiosomes failed to detect sulfate uptake (Day, unpublished data) and in this context, it should be noted that some members of the SST family, though not phylogenetically close to these candidates, can transport other metabolites in addition to sulfate, including molybdate (Tomatsu et al., 2007).

Molybdenum is an essential component of the nitrogenase enzyme. A high affinity ABC transport system encoded by *ModABC* is involved in transport of molybdate in *B. japonicum* and required for efficient nitrogen fixation (Delgado et al., 2006) suggesting that there must be transport of molybdenum across the SM. Analysis of the soybean:*B. japonicum* symbiosis in *modA or modB* mutants suggested that in addition to ModABC, a combined molybdate and sulfate transport system was also present on the bacteroid membrane.

#### Homocitrate transport

Homocitrate is essential for nitrogen-fixation, as it is a component of the iron-molybdenum (FeMo) cofactor of nitrogenase complex (Hoover et al., 1989). Isolated rhizobia rely on the plant host to supply homocitrate, as in most cases they are not able to synthesis homocitrate endogenously (Zheng et al., 1997; Hakoyama et al., 2009). Homocitrate synthase in the plant is encoded by the *FEN1* gene, and *L. japonicus fen1* mutants have reduced nitrogen fixation (Imaizumi-Anraku et al., 1997; Hakoyama et al., 2009) suggesting it is provided to bacteroids by the plant and that a transporter for homocitrate is likely to be present on the SM.

#### Metal ion transport

Iron is essential to the symbiosis as it is an integral component of proteins such as nitrogenase in the bacteroid and heme in the plant. The uptake of both ferrous and ferric iron into isolated symbiosomes has been demonstrated (Moreau et al., 1995, 1998; LeVier et al., 1996) and in soybean, GmDMT1 (Divalent Metal Transporter 1), a member of the NRAMP (Natural resistance-associated macrophage protein) family of transporters, has been identified as a ferrous iron transporter on the SM and like many other NRAMP transporters has some specificity for other metal ions including zinc, magnesium and copper (Kaiser et al., 2003). However, it is not clear whether DMT1 would transport iron into the symbiosome. Given the orientation of the symbiosome, as deduced by its development from the plasma membrane, we would expect the inside of the symbiosome to correlate with the apoplast (outside the cell). Most members of the NRAMP family transport metals into the cytoplasm and GmDMT1 was characterized by its ability to transport iron into a yeast cell. By analogy, *in situ*, GmDMT1 would transport iron out of the symbiosome, suggesting a role in regulating iron availability in the cytoplasm that is similar to *Arabidopsis* NRAMP3 and 4 (Lanquar et al., 2005) by remobilizing iron stored in the symbiosome (Udvardi and Day, 1997). However, it is possible that this transporter could be bidirectional (Kaiser et al., 2003).

*Lotus japonicus* SEN1 is a member of the Vacuolar Iron transporter (VIT1) family and is expressed specifically in nodule infected cells (Hakoyama et al., 2012). *Arabidopsis* VIT1 transports ferrous iron into the vacuole (Kim et al., 2006), analogous to import into the symbiosome. *Sen1* is essential for nitrogen fixation in *L. japonicus* suggesting it may have a role as an iron importer on the SM (Hakoyama et al., 2012). However, to date the substrate transported by SEN1 and its localization in infected cells has not been determined.

A zinc transporter, ZIP1 (Zinc regulated transporter/Iron regulated transporter-like Protein 1), has also been localized to the symbiosome and the antibodies raised against ZIP1 were able to inhibit uptake of zinc by isolated symbiosomes (Moreau et al., 2002). There has been little work on copper and manganese transport across the SM, though manganese transporters have been identified on the bacteroid membrane of a number of rhizobia and some of these result in fix^–^ phenotypes (reviewed in Udvardi and Poole, 2013).

Recently, proteins homologous to the PLAC8 superfamily, which may include metal ion transporters, were identified in the SM proteome (Clarke et al., unpublished). A subset of the PLAC8 family, known as plant cadmium resistance (PCR) proteins in plants, are a large conserved family found in fungi, algae, higher plants and animals (Song et al., 2011). They are of particular interest on the SM as two members of this family in *Arabidopsis* have been reported to play a role in the transport of heavy metals (Song et al., 2004, 2010). AtPCR1 and AtPCR2 are reported to extrude divalent cations such as cadmium and zinc from *Arabidopsis* root cells (Song et al., 2004, 2010). This would translate to an import of metal into the symbiosome and the presence of homologous proteins on the SM suggests a role in maintaining adequate nutrition for the isolated bacteroids through import of a variety of metal cations. The identification of this family as transport proteins, however, is controversial as another member of the same sub-family, FW2.2, has been shown to mediate fruit weight in tomato (Frary et al., 2000) and a role for GmFWL1 (FW2.2-like 1) in controlling nuclear size and chromatin condensation has been suggested (Libault and Stacey, 2010). However, these effects could be indirectly related to metal ion transport and compartmentalization. Clearly more work needs to done in this area to elucidate the precise role of these membrane proteins.

## CONCLUSION

The SM is the critical interface between the symbiotic partners and we still have a lot to learn about it. It is likely that a number of as yet unidentified metabolites are exchanged between the symbiotic partners in legumes. Processes that require transport of metabolites across the SM can be predicted and many putative transporters and novel integral membrane proteins have been identified on the SM through proteomic (Wienkoop and Saalbach, 2003; Catalano et al., 2004; Clarke et al., unpublished) and other approaches. Add to this the number of putative transporters with nodule specific or enhanced expression identified by studying legume transcriptomes (Benedito et al., 2010; Libault et al., 2010; Severin et al., 2010; Verdier et al., 2013) and it is clear that there is a long way to go to understand the associated metabolism and the role of transporters both on the SM and other cellular membranes in the nodule. With a more complete picture of the proteins on the SM, reverse genetic approaches can be implemented to demonstrate the phenotypes associated with disrupting particular proteins. By combining this with biochemical and biophysical assays of transport, which can now be revisited based on new genetic and molecular data available, we should be able to extend our knowledge of the transport processes required for an efficient symbiosis and perhaps identify means by which we could enhance the process and so the benefits of BNF.

## AUTHOR CONTRIBUTIONS

Victoria C. Clarke and Penelope M. C. Smith wrote the manuscript, Victoria C. Clarke, Patrick C. Loughlin, David A. Day, and Penelope M. C. Smith edited the manuscript.

### Conflict of Interest Statement

The authors declare that the research was conducted in the absence of any commercial or financial relationships that could be construed as a potential conflict of interest.
